# Effects of Time-Restricted Eating on Circadian Cortisol Secretion and Obesity-Related Metabolic Markers in Cushing’s Disease: A Pilot Study

**DOI:** 10.3390/nu18081175

**Published:** 2026-04-08

**Authors:** Lala Soltanova, Ceren Iseri, Serdar Sahin, Mihriban Kara, Suzan Aydin Guclu, Busra Yesilova, Ilkin Muradov, Banu Betul Kocaman, Pinar Kadioglu

**Affiliations:** 1 Division of Endocrinology and Metabolic Diseases, Department of Internal Medicine, Cerrahpasa Faculty of Medicine, Istanbul University-Cerrahpasa, Kocamustafapasa Street No. 53, Istanbul 34098, Türkiye; soltanova.lale92@gmail.com (L.S.); srdr_shn@hotmail.com (S.S.); i.muradov34@gmail.com (I.M.); banubetulkocaman@gmail.com (B.B.K.); 2 Department of Nutrition and Dietetic, Faculty of Health Sciences, Istanbul Kent University, Istanbul 34413, Türkiye; ceren.iseri@kent.edu.tr; 3 Pituitary Center, Istanbul University-Cerrahpasa, Istanbul 34320, Türkiye; 4 Department of Internal Medicine, Cerrahpasa Faculty of Medicine, Istanbul University-Cerrahpasa, Istanbul 34320, Türkiye; mihribankara903@gmail.com; 5 Division of Endocrinology, Metabolism and Diabetes, Department of Internal Medicine Nursing Service, Cerrahpasa Faculty of Medicine, Istanbul University-Cerrahpasa, Istanbul 34320, Türkiye

**Keywords:** Cushing’s disease, time-restricted eating, intermittent fasting, circadian cortisol

## Abstract

**Purpose:** The aims of this study were to evaluate the feasibility of time-restricted eating (TRE) in patients with Cushing’s disease (CD) and assess its effects on body weight and metabolic parameters. **Methods:** Twelve CD patients in remission with obesity were enrolled in a TRE program restricting food intake to 10:00–18:00. Anthropometric data, glycemic and lipid profiles, and circadian cortisol secretion were assessed at baseline and post-intervention. Serum cortisol levels were measured at multiple time points to evaluate diurnal patterns. **Results:** Nine patients completed the study. Over the 12-week period, participants showed a significant reduction in body weight, with median values decreasing from 93.8 kg [83.1–106.5] to 82.6 kg [76.9–100.3] (*p* = 0.011). Body mass index (BMI) also declined from 37.6 kg/m^2^ [34.2–39.7] to 34.4 kg/m^2^ [32.6–38.3] (*p* = 0.012). No statistically significant changes were observed in fasting glucose, HbA1c, or lipid parameters. Notably, 24 h urinary free cortisol levels significantly decreased (*p* = 0.01), and serum cortisol showed a downward trend at all measured time points, with the most pronounced reductions during mid-day and evening hours. No clinical or biochemical evidence of CD relapse was observed during the 12-month follow-up. **Conclusions:** Time-restricted eating is a feasible and well-tolerated dietary approach for patients with CD in remission, promoting weight loss and modest improvements in metabolic markers and cortisol rhythmicity.

## 1. Introduction

Cushing’s disease (CD) is an endocrine disorder characterized by excessive secretion of adrenocorticotropic hormone (ACTH), most commonly due to a pituitary adenoma, which in turn stimulates increased cortisol production by the adrenal glands [1]. Cushing’s syndrome refers to hypercortisolism due to any cause, whereas Cushing’s disease is a specific form of Cushing’s syndrome caused by an adrenocorticotropic hormone (ACTH)-secreting pituitary adenoma [2]. Chronic hypercortisolemia leads to a wide spectrum of metabolic and systemic complications, including hypertension, type 2 diabetes mellitus, dyslipidemia, central obesity, osteoporosis, and neuropsychiatric disturbances [3,4,5,6,7,8,9]. Obesity is one of the most prevalent and challenging features of CD. While the reported prevalence of CD among obese individuals ranges from 0.23% to 9.3%, the prevalence of obesity among patients with CD is higher, estimated between 70% and 85% [10,11,12,13]. A major challenge in the management of CD is the persistence of obesity and cardiovascular risk, even after clinical and biochemical remission [14]. Previous studies have shown that despite achieving remission, 38% of patients with CD continue to exhibit obesity. Although the prevalence of obesity may initially decrease to 31% following successful transsphenoidal surgery, an increase has been observed, reaching up to 44.8% within the first year of surgery. These findings underscore the persistent challenge of weight management in this patient population. In recent years, our clinical observations have suggested that, in addition to classical central obesity, some patients with CD may also present with a simple (generalized) obesity phenotype. This evolving clinical pattern highlights the need to broaden our understanding of obesity presentations in CD [6,15,16]. Despite the considerable burden of obesity, there remains no universally accepted or standardized strategy for its management in these patients. Although remission can be achieved through medical or surgical interventions, attaining sustained and meaningful weight loss remains difficult, and data on long-term weight outcomes following remission are currently limited in the literature.

The initial step in the treatment of obesity typically involves a structured and comprehensive lifestyle intervention program aimed at weight loss. Such programs generally consist of individualized nutritional therapy, regular physical activity, and behavioral modifications. Time-restricted eating (TRE), a form of intermittent fasting, has been explored as a potential strategy for weight management in the general population, particularly among individuals with overweight or obesity, with evidence suggesting metabolic benefits related to energy deficit and circadian rhythm alignment [17]. This approach involves limiting food intake to specific hours of the day. TRE has been studied across various populations, including individuals with obesity, metabolic syndrome, type 2 diabetes, and polycystic ovary syndrome [18,19,20]. It has been associated with modest weight loss, improvements in insulin sensitivity, reductions in fasting glucose and blood pressure, and favorable changes in lipid profiles. Aligning the eating window of TRE with the physiological circadian rhythm of cortisol secretion may help synchronize meal timing with endogenous hormonal fluctuations and potentially improve metabolic parameters independent of weight loss in patients with CD.

To our knowledge, no previous studies have specifically investigated time-restricted eating (TRE) in patients with Cushing’s disease (CD). Therefore, we aimed to assess the feasibility of this dietary approach and examine its associations with dietary adherence, weight loss, and metabolic changes.

## 2. Materials and Methods

### 2.1. Participants

A total of 224 patients with CD who were followed up at the Division of Endocrinology-Metabolism and Diabetes, Istanbul University-Cerrahpasa, Cerrahpasa Faculty of Medicine, between 2004 and 2024 were screened in the study. All participants had previously achieved remission of hypercortisolism and were eucortisolemic at the time they were contacted and enrolled in the study. These patients had reported difficulty losing weight during routine outpatient clinic visits, which was documented in their clinical record. The inclusion criteria were (i) age ≥ 18 years; (ii) patients diagnosed with CD who underwent surgical treatment; (iii) patients in remission following surgical or medical therapy; and (iv) Cushing’s patients with obesity (BMI > 30 kg/m^2^).

Exclusion criteria included (i) recent pituitary surgery within the past 6 months; (ii) pregnancy; (iii) current breastfeeding; and (iv) Patients with active CD.

**Figure 1:**
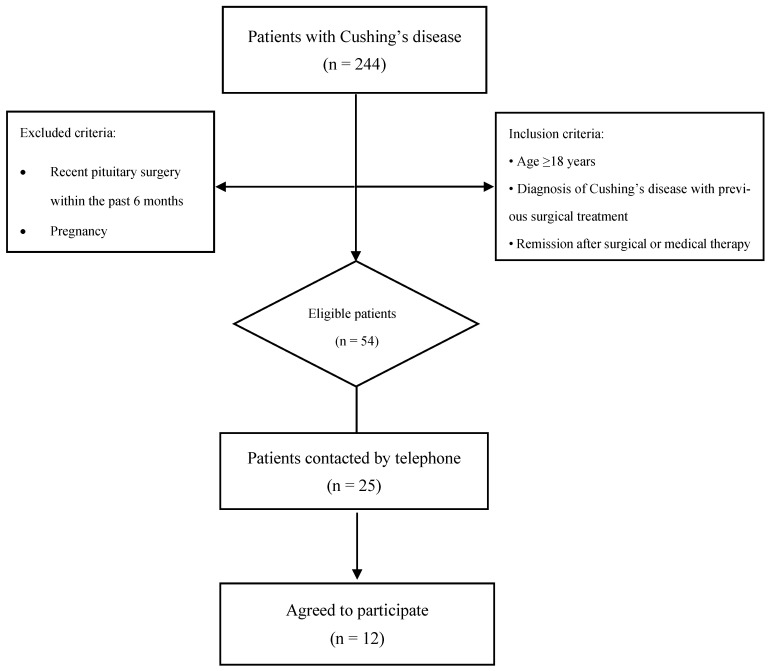
Flowchart of patient selection and inclusion in the time-restricted eating study in patients with Cushing’s disease.

Among the 224 patients with Cushing’s disease screened, 54 met the inclusion criteria and were regularly attending follow-up visits. From this eligible cohort, 25 patients were consecutively contacted using the available contact information in our records. These represented patients with valid and unchanged phone numbers who could be reached and responded to the call. Of these 25 patients, 12 agreed to participate in the dietary intervention (Figure 1).

Written informed consent was obtained from all participants. The Ethics Committee of Istanbul University-Cerrahpasa, Cerrahpasa Faculty of Medicine, approved the study protocol (E-83045809-604.01-1234916, 19 February 2025). All procedures were performed in accordance with the ethical standards of the Declaration of Helsinki.

### 2.2. Endocrinological Definitions and Follow-Up Protocols

The diagnosis of CD was established according to the international consensus guidelines for the diagnosis and management of CD (2021) and the consensus recommendations on Cushing’s syndrome [21,22]. Active CD was defined as the presence of elevated late-night salivary cortisol (LNSC) and urinary free cortisol (UFC) levels, accompanied by clinical signs of hypercortisolism in patients with an adrenocorticotropic hormone (ACTH)-secreting pituitary adenoma. Remission was defined as the improvement or resolution of clinical symptoms following surgery, along with suppressed serum cortisol levels after dexamethasone administration (<1.8 μg/dL), and normalization of 24 h UFC and LNSC [23,24]. In patients with confirmed recurrence or residual tumors, hypercortisolemia was confirmed to be due to ACTH excess [25]. Medical therapy was initiated in patients who were not suitable candidates for reoperation. Prednisolone or hydrocortisone replacement therapy was started in patients diagnosed with central adrenal insufficiency, defined as a serum cortisol level < 2 μg/dL either immediately after transsphenoidal surgery (TSS) or within 48 h postoperatively [24]. Cortisol, ACTH, and UFC levels were measured using the electrochemiluminescence immunoassay method.

### 2.3. Anthropometric Measurements

Anthropometric measurements were performed with participants wearing light clothing and no shoes. Body weight, body mass index (BMI), fat percentage, and muscle mass were assessed using a segmental bioelectrical impedance analyzer (Tanita BC-418 Corporation, Tokyo, Japan). Waist circumference was measured with a flexible, non-compressive tape placed directly on the skin at the midpoint between the lower costal margin and the iliac crest. Hip circumference was measured at the widest point over the buttocks. All anthropometric assessments were conducted at baseline (week 0) and after 12 weeks of intervention.

### 2.4. Study Protocol

Serum cortisol levels were measured at multiple time points to assess postprandial and diurnal changes. Based on previous evidence showing that cortisol levels rise approximately 30 min after meal intake, we took postprandial samples accordingly [26]. Patients were hospitalized for three days and two nights. No blood samples were collected on the first day to allow adaptation to the hospital environment. Serum cortisol measurements were initiated on the second morning. After an 8 h overnight fast, the first basal blood sample was obtained before breakfast. Fasting and postprandial serum samples were then collected at predetermined intervals: before and 30 min after breakfast; before and 30 min after lunch; before and 30 min after dinner; midnight and again the following morning after an overnight fast. Following the 12-week dietary intervention, patients resumed their routine outpatient follow-up care. Blood samples were transported to the laboratory immediately after collection and were analyzed on the same day according to standard laboratory procedures.

### 2.5. Dietary Protocol

Following the clinical evaluations conducted at the Endocrinology outpatient clinic, all patients were referred for a dietitian assessment. The energy requirements of each patient were calculated considering age, sex, height, weight, and lifestyle habits. A mildly energy-restricted diet was prescribed for each patient, with daily caloric intake reduced by approximately 500 kcal below their total energy requirements [27]. Within the dietary intervention, a time-restricted feeding window between 10:00 and 18:00 was implemented. Meal times were planned as breakfast at 10:00, lunch between 12:00 and 14:00, and dinner finished by 18:00 at the latest. Outside of this time window, only non-caloric beverages (e.g., water, unsweetened herbal teas) were permitted. Follow-up visits were conducted face-to-face at weeks 0, 4, 8, and 12, and via remote consultations at weeks 2, 6, and 10. Prior to each visit, all participants were asked to provide 3-day dietary intake records. During remote consultations, participants were interviewed regarding fasting duration, meal timing, and dietary intake.

## 3. Statistical Analysis

The analysis was conducted using the Statistical Package for the Social Sciences (SPSS), version 26.0 (IBM Corp., Armonk, NY, USA). The distribution of variables was assessed using the Kolmogorov–Smirnov test. Continuous variables with non-normal distribution are presented as median [interquartile range, IQR], while categorical variables are expressed as frequencies and percentages. Comparisons between baseline and post-intervention measurements were performed using the Wilcoxon signed-rank test for paired samples. Data visualization was performed using boxplots and line graphs to illustrate trends in anthropometric and biochemical parameters over time. A two-sided *p*-value of <0.05 was considered statistically significant, and the confidence level was set at 95%.

## 4. Results

Of the 12 patients initially assessed, 11 patients commenced the dietary intervention and 9 completed the 12-week intermittent fasting program. Among the 9 patients with CD who completed the study, 8 (88%) were female and 1 (12%) was male. Sociodemographic data and current medication use of the patients are shown in Table 1.

**Figure 2:**
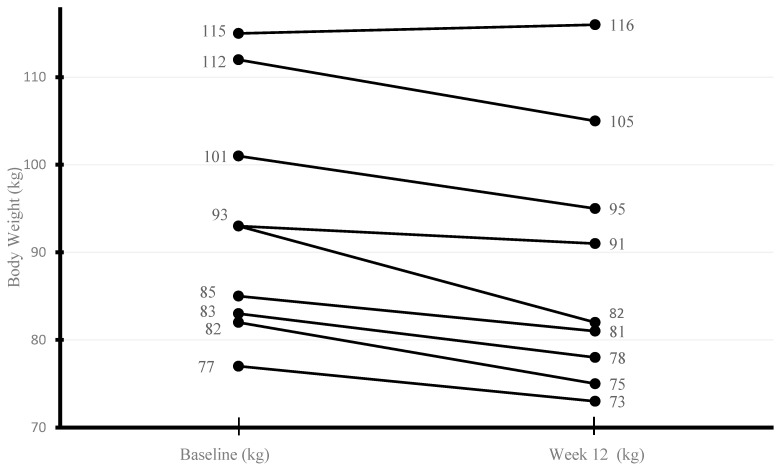
Individual patient body weights before and after 12 weeks of time-restricted eating. Each line represents one patient.

Over the 12-week period, participants exhibited a notable decrease in body weight, with median values dropping from 93.8 kg [83.1–106.5] to 82.6 kg [76.9–100.3] (*p* = 0.011) (Figure 2). A parallel downward shift was observed in BMI, which declined from 37.6 kg/m^2^ [34.2–39.7] to 34.4 kg/m^2^ [32.6–38.3] (*p* = 0.012), suggesting a consistent pattern of improvement in overall adiposity. Waist circumference followed a similar trajectory, narrowing from a median of 109 cm [106.5–117] to 104 cm [102–108] (*p* = 0.008), reflecting central fat reduction. In contrast, hip circumference remained relatively stable across the study period, with minimal variation between baseline and week 12 measurements (Table 2). Consistent with these findings, eight patients with CD experienced weight loss, while one patient exhibited no change in body weight.

Fasting plasma glucose decreased from a median of 100 mg/dL [90–125] at baseline to 98 mg/dL [85–113] at week 12 (*p* = 0.058), approaching statistical significance. HbA1c levels remained unchanged at 5.7% [5.4–7.8] at baseline and 5.7% [5.3–7.2] at week 12 (*p* = 0.12). LDL cholesterol decreased from 123.1 mg/dL [94.5–158.9] to 115 mg/dL [88.5–151.4], while triglyceride levels declined from 155 mg/dL [99–164] to 139 mg/dL [86–146]. In contrast, total cholesterol slightly increased from 187 mg/dL [159–235] to 191 mg/dL [148–228], and HDL cholesterol increased from 49 mg/dL [42–58] to 52 mg/dL [44–60]. However, none of these changes reached statistical significance.

Similarly, the median cortisol concentration after the overnight 1 mg DST showed a non-significant decrease, from 1.4 µg/dL [1.1–1.7] at baseline to 1.3 µg/dL [1.12–1.47] at week 12 (*p* = 0.18). By contrast, 24 h UFC levels declined significantly, from 40 µg/24 h [18.6–96.9] at baseline to 16.8 µg/24 h [12.5–33.2] at week 12 (*p* = 0.01) (Table 3). Over the course of the intervention, a gradual yet consistent reduction in cortisol levels was observed across all time points.

**Figure 3:**
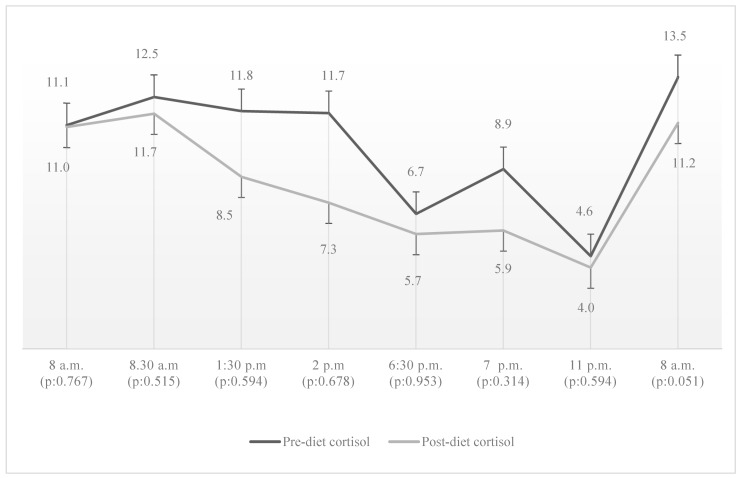
Diurnal serum cortisol profile before and after 12 weeks of time-restricted eating in patients with Cushing’s disease in remission.

Median fasting cortisol concentrations measured before breakfast decreased slightly from 11.18 µg/dL to 11.01 µg/dL (*p*: 0.767), while post-breakfast values showed a more discernible decline from 12.57 µg/dL to 11.67 µg/dL (*p*: 0.515). At lunchtime, fasting cortisol levels fell from 11.87 µg/dL to 8.54 µg/dL (*p*: 0.594), accompanied by a post-lunch decrease from 11.73 µg/dL to 7.25 µg/dL (*p*: 0.678). Evening measurements also followed this downward trajectory: fasting cortisol dropped from 6.71 µg/dL to 5.70 µg/dL (*p*: 0.953), and post-dinner levels fell from 8.92 µg/dL to 5.88 µg/dL (*p*: 0.314). Midnight serum cortisol values declined modestly, from 4.60 µg/dL to 4.04 µg/dL (*p*: 0.594). This trend persisted into the next day, with fasting cortisol the following morning decreasing from 13.48 µg/dL to 11.21 µg/dL (*p*: 0.051) (Figure 3).

Three participants discontinued the study. One patient moved to another city and could not attend the planned hospitalization. One patient stopped the diet due to a family bereavement, and another withdrew because of a work-related change requiring frequent international travel and irregular schedules. No adverse events related to the intervention were reported.

Following three months of dietary intervention, one patient demonstrated improved glycemic control, allowing for a reduction in oral antidiabetic therapy from three agents to two. In a different patient receiving medical therapy for hypercortisolism, the daily dose of metyrapone was successfully reduced from 750 mg/day to 500 mg/day. Notably, no patient required an escalation in the dosage of their Cushing’s disease-specific treatment either during the intervention period or throughout the subsequent one-year follow-up.

## 5. Discussion

The TRE regimen was a well-tolerated and effective model for patients with CD in remission. This dietary approach was associated with notable improvements in anthropometric parameters, including reductions in body weight, body mass index, and waist circumference. Biochemical assessment revealed a decrease in cortisol burden, as evidenced by lower values in the overnight 1 mg DST and 24 h UFC excretion. The observed decline in 24 h cortisol levels indicates a probable enhancement in hypothalamic–pituitary–adrenal (HPA) axis regulation. No clinical or biochemical relapse of CD was observed during the one-year follow-up after the three-month intervention, suggesting that time-restricted eating was well tolerated in this cohort.

TRE has generally shown beneficial effects on anthropometric parameters, even though data specifically focused on patients with CD are scarce. Gabel et al. demonstrated that a 12-week TRE intervention in 23 obese individuals resulted in significant reductions in body weight and BMI [28]. A recent systematic review further supported these findings, showing that short-term TRE interventions (1–12 weeks) in obese men and women led to body weight reductions ranging between 1% and 4% [29]. In our study, a 12-week dietary intervention of comparable duration to previous studies was implemented in patients with CD in remission. Although the reduction in fat percentage was not statistically significant, it was comparable to values reported in previous studies. The more pronounced decreases in BMI and body weight may be attributed to reduced opportunities for snacking and late-night eating, as well as a slight caloric deficit resulting from the restriction of food intake to a specific time window.

TRE and ketogenic diets have both been shown to produce beneficial effects on fasting glucose and cholesterol levels in individuals with diabetes, hypertension, and obesity [30,31]. Parr et al. compared an extended feeding schedule with a time-restricted feeding regimen (10:00–17:00). After a 10-day run-in period followed by a 5-day intervention, time-restricted feeding was found to be better tolerated and associated with improvements in glucose and insulin responses [32]. In a study by Guarnotta et al., patients with active CD underwent a 3-week very-low-calorie ketogenic diet followed by a 2-week low-calorie, carbohydrate-restricted regimen. After this 5-week intervention, notable improvements were observed in LDL, triglyceride, and HDL levels, while the reduction in fasting glucose was relatively modest [33]. Additionally, reductions in the 1 mg overnight DST and 24 h UFC levels were minimal. In contrast, our study implemented a 12-week TRE regimen in patients with CD in remission. The absence of pronounced reductions in LDL cholesterol, triglycerides, and fasting glucose may be related to the biochemical remission status of the participants, the small sample size, the lack of severe caloric or carbohydrate restriction, and the fact that baseline metabolic parameters were already within the upper-normal or borderline elevated range. Given that the patients in Guarnotta’s study had active disease, the hormonal changes observed are more likely to reflect the metabolic effects of the ketogenic diet than a direct influence on the underlying pathophysiology of CD. In our cohort, the observed decrease in 24 h UFC levels may instead indicate a more responsive HPA axis during remission.

The relationship between fasting and cortisol secretion appears to be complex, depending on both the duration and intensity of the fasting state. Previous studies have reported different results. For instance, a study involving eight healthy men demonstrated an increase in cortisol levels following a 5-day fasting period, suggesting a metabolic stress response [34]. In contrast, Magyar et al. observed a decrease in cortisol levels after 48 h of fasting in 20 healthy female participants [35]. These findings suggest that sex, metabolic state and variability in fasting protocols may affect the direction and magnitude of cortisol responses. Furthermore, a 2016 meta-analysis concluded that fasting tends to increase cortisol levels, whereas calorie-restricted diets do not appear to have this effect [36]. In our study, patients who adhered to a time-restricted and mildly calorie-restricted dietary regimen showed a numerical decrease in morning cortisol levels after 12 weeks; however, this change did not reach statistical significance, possibly due to the small sample size. This finding suggests that the 16 h fasting period did not impose sufficient physiological stress to trigger hypercortisolemia, thereby supporting the potential suitability of this dietary strategy in patients with CD. Analysis of the 24 h circadian cortisol rhythm revealed a preserved diurnal pattern following the dietary intervention. However, a numerical decrease in serum cortisol levels was observed, with the most pronounced reductions occurring during midday and evening hours. Although these changes did not reach statistical significance, they may indicate a trend toward attenuation of hypercortisolemia and a subtle improvement in cortisol rhythmicity. Nevertheless, the fasting conditions and sampling protocols in our study differed from those reported in previous studies. Earlier studies evaluated cortisol responses after prolonged fasting periods (48 h or up to 5 days) with frequent sampling intervals, whereas our measurements were obtained following a 16 h fasting period within a time-restricted eating regimen. Therefore, cortisol dynamics similar to those described in prolonged fasting studies cannot be completely excluded.

One possible explanation for the observed trend toward lower cortisol levels may be the improved alignment between feeding time and the physiological circadian rhythm of cortisol secretion. Cortisol follows a well-established diurnal pattern, characterized by higher levels in the early morning and a gradual decline toward the evening. Time-restricted eating, particularly when the eating window is limited to daytime hours, may reinforce this natural circadian organization of metabolic and endocrine processes. By avoiding late-evening caloric intake and concentrating food consumption earlier in the day, TRE may reduce circadian misalignment and support a more physiological pattern of HPA axis activity. Thus, rather than acting as a direct suppressor of cortisol secretion, TRE may promote a more physiological circadian regulation of cortisol dynamics.

In the study cohort, there was one patient who did not experience any weight loss during the intervention period. In this patient, remission of CD was confirmed during the three-month follow-up. Even after subsequent initiation of GLP-1 receptor agonist therapy outside the study protocol, no significant weight loss was observed during the following three-month period. A detailed medical history revealed that the patient had been consistently struggling with excessive weight and had shown only limited weight loss even after curative surgery for CD. We also learned that the patient’s sister had undergone bariatric surgery due to obesity. It remains unclear whether the patient fully adhered to other components of obesity management, such as regular exercise and lifestyle changes. Taken together, these findings raise the possibility that both suboptimal adherence to non-pharmacological treatment strategies and long-term epigenetic effects of hypercortisolism may contribute to ongoing metabolic dysregulation, even after biochemical remission [37].

This study has several limitations that warrant consideration. First, the sample size is relatively small, which may restrict the generalizability of the findings. Second, the absence of a control group prevents a direct comparison between the dietary intervention and standard care or alternative dietary approaches. Third, adherence to the time-restricted eating protocol was primarily assessed through patient self-reports during follow-up visits, and no objective monitoring tools were used to verify compliance. Finally, because the TRE protocol was implemented together with a modest caloric restriction, the independent effects of meal timing could not be clearly distinguished from those of reduced energy intake.

## 6. Conclusions

In this study, we demonstrated that time-restricted eating is an effective dietary intervention for promoting weight loss in patients with CD in remission. Beyond its favorable impact on weight reduction, TRE was also associated with improvements in markers of hypercortisolemia. These findings suggest that TRE may represent a feasible and beneficial strategy for managing metabolic complications in post-remission CD patients. However, further studies involving larger cohorts and extended follow-up periods are necessary to confirm these findings and to evaluate the long-term sustainability and endocrine effects of TRE in this population.

## Figures and Tables

**Table 1:** Clinical and sociodemographic characteristics and current medications of the study participants (*n* = 9).

Patient	Age/Sex	Education	Marital Status	Employment	Smoking	Alcohol	Current Medications
1	63/F	<12 yrs	Married	Retired	Non-smoker	No	Pasireotide 0.9 mg; Metformin/Sitagliptin 50/1000 mg; Empagliflozin 10 mg; Atorvastatin 10 mg; Levothyroxine 100 mcg
2	38/F	<12 yrs	Married	Employed	Smoker	No	Prednisolone 7.5 mg; Sitagliptin 100 mg; Ramipril 5 mg; Atorvastatin 10 mg
3	62/F	<12 yrs	Married	Unemployed	Non-smoker	No	Telmisartan 40 mg; Vildagliptin/Metformin 50/1000 mg; Empagliflozin 25 mg
4	61/F	<12 yrs	Married	Unemployed	Non-smoker	No	Vildagliptin/Metformin 50/1000 mg; Insulin glargine 14 U; Empagliflozin 25 mg; Nifedipine 30 mg; Olmesartan 20 mg; Furosemide 40 mg; Venlafaxine 37.5 mg; Atorvastatin 10 mg
5	47/F	<12 yrs	Married	Employed	Non-smoker	No	Candesartan 16 mg
6	46/F	≥12 yrs	Married	Employed	Non-smoker	No	Levothyroxine 125 mcg; Pantoprazole 40 mg
7	70/F	<12 yrs	Divorced	Retired	Non-smoker	No	Lercanidipine 10 mg; Indapamide 1.5 mg; Ramipril 5 mg
8	53/F	<12 yrs	Married	Unemployed	Non-smoker	No	Metyrapone 750 mg; Metformin 1000 mg; Candesartan/Hydrochlorothiazide 16/12.5 mg
9	28/M	≥12 yrs	Married	Employed	Smoker	No	None

F: female, M: male. yrs: years.

**Table 2:** Anthropometric and Body Composition Parameters at Baseline and Week 12 in Patients with Cushing’s Disease.

	Patients with Cushing’s Disease		*p*
	*n* = 9		
	Baseline Median [IQR]	Week 12	
Weight (kg)	93.8 [83.1–106.5]	82.6 [76.9–100.3]	0.011
BMI (Kg/m^2^)	37.6 [34.2–39.7]	34.4 [32.6–38.3]	0.012
Fat (%)	44.1 [40.5–45.8]	41.2 [38.5–43.6]	0.173
Muscle Mass (kg)	46.7 [44.3–51.9]	45.8 [43.3–52.2]	0.767
Waist circumference (cm)	109 [106.5–117]	104 [102–108]	0.008
Hip circumference (cm)	116 [112–124]	117 [108–121]	0.108

BMI: Body Mass Index, IQR: Interquartile Range.

**Table 3:** Metabolic Parameters at Baseline and Week 12 in Patients with Cushing’s Disease.

	Baseline Median [IQR]	Week 12	*p*
Fasting Plasma Glucose [mg/dL]	100 [90–125]	98 [85–113]	0.058
HbA1c [%]	5.7 [5.4–7.8]	5.7 [5.3–7.2]	0.12
LDL [mg/dL]	123.1 [94.5–158.9]	115 [88.5–151.4]	0.31
Total Cholesterol [mg/dL]	187 [159–235]	191 [148–228]	0.55
HDL [mg/dL]	49 [42–58]	52 [44–60]	0.28
Triglycerides [mg/dL]	155 [99–164]	139 [86–146]	0.51
Overnight 1 mg DST [µg/dL]	1.4 [1.1–1.7]	1.3 [1.12–1.47]	0.18
Urinary free cortisol [µg/24]	40 [18.6–96.9]	16.8 [12.5–33.2]	0.01

Reference ranges: Fasting Plasma Glucose, 70–125 mg/dL; HbA1c, 4.8–6%; LDL cholesterol, <100 mg/dL; Total cholesterol, 50–200 mg/dL; HDL cholesterol, >60 mg/dL; triglycerides, <200 mg/dL; Urinary free cortisol 11.5–102 µg/24. HbA1c: glycated hemoglobin A1c, LDL: Low-Density Lipoprotein, HDL: High-Density Lipoprotein, DST: Dexamethasone Suppression Test.

## Data Availability

The datasets generated and analysed during the current study are not publicly available due to ethical and patient confidentiality restrictions, but are available from the corresponding author upon reasonable request.
